# Frontiers in Age-Related Diseases and Nutritional Interventions

**DOI:** 10.3390/nu17101658

**Published:** 2025-05-13

**Authors:** Caiziyu Yu, Lili Qiu, Xiaoyu Wang

**Affiliations:** 1 College of Food Science & Nutritional Engineering, China Agricultural University, Beijing 100083, China; yuczy@cau.edu.cn (C.Y.); qiulily980829@126.com (L.Q.); 2 Key Laboratory of Precision Nutrition and Food Quality, Department of Nutrition and Health, China Agricultural University, Beijing 100193, China

## 1. Introduction

The aging population presents one of the greatest challenges to sustainable development, progressing at an unprecedented rate in recent years. Aging involves an irreversible, progressive decline in physiological functions, ultimately leading to a range of age-related diseases, including neurodegenerative, cardiovascular, gastrointestinal, and immune system diseases. Over the years, extensive efforts have been devoted to investigating the biology of aging and the mechanisms underlying age-associated degenerative diseases, with the goal of developing strategies to alleviate age-related health issues. A growing body of research has highlighted the significant role of diet in regulating aging and the development of age-associated diseases. This Special Issue of *Nutrients*, titled “Dietary Strategies for Prevention of Geriatric Diseases and Exploring the Mechanism of Aging”, aims to present dietary and nutritional strategies for alleviating aging and age-related degenerative diseases in the elderly population, while also advancing our knowledge of the underlying mechanisms of and age-related pathology. The studies featured in this Special Issue can be categorized into the following two main areas: (i) Aging and Age-related Diseases, and (ii) Nutritional Interventions for the Elderly. Together, these two themes offer new insights and practical approaches to understanding and managing age-related health issues, paving the way for more effective strategies to improve the quality of life among the elderly and promote healthy aging.

## 2. Aging and Age-Related Diseases

One article explored the factors influencing the cellular and molecular hallmarks of aging. Reduced telomere length and various epigenetic alterations (DNA methylation, histone modifications, and chromatin remodeling) are among the key cellular and molecular markers of aging. Additionally, S. Liu et al. investigated the causal relationship between the intake of different types of meat and biological aging [1]. Their findings revealed a positive causal relationship between the intake of meat and accelerated DNA methylation PhenoAge. Moreover, processed meat consumption was negatively associated with telomere length, suggesting that increased meat intake may accelerate the biological aging process.

In addition, several studies in this Special Issue focused on age-related degenerative diseases. Although each study addressed a different topic, their findings collectively provide further insight into the mechanisms and contributing factors of such diseases. C. Chang et al. identified a significant association between nutritional risk and dynapenia, specifically in elderly people aged 75 years and above, suggesting that age may moderate the relationship between nutritional status and dynapenia in older populations [2]. A comprehensive review authored by W. Yu et al. summarized the alterations in the immune system associated with aging and elucidated the mechanisms of immunosenescence [3]. Aging impairs both innate and adaptive immunity; this decline in immunity not only exacerbates the aging process but also accelerates the onset of infectious diseases, autoimmune disorders, and cancer. The study by L. Gaussens et al. examined the associations between vitality components (appetite loss and weight loss) and other domains of Intrinsic Capacity (IC) in the elderly, such as cognition, vision, hearing, psychology and locomotion [4]. Appetite and weight loss, both common among the elderly, were found to be associated with other potential IC impairments, i.e., psychological and locomotor functions. In another study, X. Sang et al. explored the changes in physicochemical properties of the colonic mucus layer with increasing age and the underlying mechanisms [5]. In aging mice, changes in the content and structure of mucin were found to be linked to impaired mucus barrier function. Additionally, decreased expression of the genes that typically regulate goblet cell formation resulted in a reduction in the proportion of goblet cells and mucin content, while the low expression of mucin glycosylase led to shorter O-glycan chains, affecting mucus microstructure and thus reducing mucus barrier function with aging. Together, these studies provide valuable insights into the mechanisms related to aging by exploring the diverse physiological changes in the body during the aging process and the various factors that trigger age-related diseases.

## 3. Nutritional Interventions for the Elderly

Dietary interventions have been proven to be among the most powerful and consistent approaches to mitigating age-related diseases and dysfunction. The prevention and treatment of aging-induced diseases can be achieved through proper dietary habits, nutritional supplements, and adequate nutrient intake.

Two papers in this Issue provide significant insights into dietary intervention strategies. Through latent class trajectory analysis, C. Zhang et al. discovered that Chinese older adults with a dietary variety score (DVS) change trajectory characterized as “Moderate–Slow decline–Slow growth” were at an increased risk of frailty [6]. Their findings suggest that maintaining a high dietary diversity trajectory could reduce the risk of frailty in older Chinese adults, while the optimal time for intervention is in the early stages of aging. In addition, H. -Y. Lee et al. presented a review on the interplay between dietary restriction-related molecular signaling proteins, signaling pathways, lipid metabolism, and aging. The review examined diet-induced modifications in cell membrane lipids and commensal bacteria-driven shifts in lipid metabolism, ultimately highlighting the pivotal role of lipid metabolism in the aging process [7]. 

Among the articles related to specific nutrient-based intervention strategies for aging, Q. Chen et al. conducted the first comprehensive assessment of the relationship between the intake of methyl donor nutrients (MDNs) and cognitive function in the elderly. They found that MDNs may have the potential to enhance cognitive function by regulating microbial homeostasis [8]. In addition, C. Cochet et al. conducted a systematic review of the existing evidence on the efficacy of nutritional supplementation in supporting muscle and mitochondrial health in elderly individuals with sarcopenia or malnutrition [9]. The review concluded that branched-chain amino acids, in association with vitamin D, may help in treating sarcopenia and enhancing mitochondrial bioenergetics and redox activity. Based on these findings, the authors proposed dietary recommendations for treating sarcopenia in older people. In a related study, O. Ezaki summarized three potential extracellular signals that could improve muscle mass and function through the supplementation of medium-chain triglycerides (MCT; C8:0 and C10:0) during meals in frail older adults. They provide guidance for future clinical trials evaluating the use of MCT in treating primary (age-related) sarcopenia [10]. S. Wang et al. evaluated the efficacy and safety of exogenous nucleotides as aging supplements for the elderly [11], finding that nucleotide intervention may be a promising strategy due to its anti-aging effects and ability to enhance the quality of life in older adults. In another review, W. Yu et al. emphasized the importance of counteracting immunosenescence through the use of specific nutrients, such as macronutrients including proteins, fatty acids (e.g., short-chain fatty acids), and micronutrients including vitamins (A, D, E), minerals (e.g., zinc), as well as probiotics and prebiotics [3]. A review by N.A. Rivero-Segura et al. explored the potential of nutraceuticals—such as vitamins, polyphenols, flavonoids, saponins, fatty acids, probiotics, and plant extracts—in preventing age-related diseases by targeting biological markers of aging (such as telomere shortening, epigenetic changes, mitochondrial dysfunction, etc.) and emphasized their role as natural anti-aging agents [12]. Overall, these articles present viable strategies for alleviating aging and age-associated diseases through specific supplements and nutrients.

## 4. Conclusions

This Special Issue of *Nutrients* sheds light on the underlying mechanisms related to aging and age-associated pathology, while also exploring optimal nutritional strategies for the prevention and mitigation of geriatric diseases. The aging process and associated decline in physiological functions pose substantial challenges to the health and quality of life of the elderly. Several studies included here emphasize the potential of dietary and nutritional interventions to counteract these age-related alterations. Collectively, the papers published in this Special Issue underscore the significant role of dietary and nutritional interventions in addressing the multifaceted challenges of aging and age-related diseases. In summary, this Special Issue serves as a platform to enhance our understanding of the biological factors and pathogenic mechanisms involved in aging and age-related diseases, offering meaningful insights into dietary approaches for the prevention and management of geriatric diseases.
